# Supplementary material for: Content-rich biological network constructed by mining PubMed abstracts
Source: BMC Bioinformatics. 2004 Oct 8;5:147. doi: 10.1186/1471-2105-5-147 (PMC528731; doi:10.1186/1471-2105-5-147)
Supplement: Additional File 5 — The original Chilibot query results of the term "long-term potentiation (LTP)" and 22 other terms, limiting the latest references analyzed to the years 1990, 1995, 2000, and 2004. [file 1471-2105-5-147-S5.bz2 › chilibotAdditionalFile5/ltp2000/html/LTP_PKC.html]

 


 **LTP** and **PKC** 
  
Found 149 abstracts in PubMed,  **30 abstracts were retrieved and analyzed**.  


---

 Search Google  |
 PDF files only 
|  EDU domain only 

---

**Interactive relationship** (e.g. stimulation, inhibition, etc)

- It is concluded that induction of anoxic  **LTP**  requires the activation of postsynaptic  **PKC** .  Ref: 9855311 Neuroreport, 1998
- We suggest that neither  **PKC**  nor PKA is required to induce  **LTP**  at this synapse.  Ref: 11069602 Eur J Neurosci, 2000
- However,  **PKC**  is involved in the regulation of  **LTP**  induction, via the molecular switch process.  Ref: 11069602 Eur J Neurosci, 2000
- Superfusion of the hippocampal slices with the protein kinase C  **PKC**  inhibitors NPC 15437 20 microM or H 7 20 microM specifically prevented the induction of anoxic  **LTP** .  Ref: 9855311 Neuroreport, 1998
- Furthermore, the  **LTP**  NMDA deficit in mGluR5 mutant mice could be rescued by stimulating protein kinase C  **PKC**  with 4beta phorbol 1 3 dibutyrate PDBu.  Ref: 10454358 Learn MemLearn Mem, 1998
- These data show that in  **LTP**  K the in situ phosphorylation of pre and postsynaptic  **PKC**  substrates is increased, indicating that NMDA receptor dependent and NMDA receptor independent  **LTP**  share common calcium dependent expression mechanisms, including activation of pre and postsynaptic  **PKC** .  Ref: 10822151 Neurosci Lett, 2000
- The possibility that protein kinase C  **PKC**  is involved in the induction of N methyl D aspartate NMDA receptor dependent long term potentiation  [ **LTP** ]   **LTP**  at CA1 synapses in the hippocampus has been the subject of considerable investigation.  Ref: 11069602 Eur J Neurosci, 2000
- Our results demonstrate that the gamma isotype of  **PKC**  is not essential for phorbol ester mediated synaptic facilitation, and place limitations on the possible roles of  **PKC**  in  **LTP** .  Ref: 10456088 Learn MemLearn Mem, 2000
- Mice deficient in  **PKC**  beta showed normal brain anatomy and normal hippocampal synaptic transmission, paired pulse facilitation, and long term potentiation  [ **LTP** ]  and normal sensory and motor responses.  Ref: 10934237 J Neurosci, 2000
- It has been shown previously that mice lacking the brain specific gamma subtype of  **PKC**  display abnormal long term potentiation  [ **LTP** ]   **LTP** .  Ref: 10456088 Learn MemLearn Mem, 2000
- These results indicate that postsynaptic CaM KII and  **PKC**  activities are essential for maintaining  **LTP**  expression, but CaN activity limits synaptic strength at stable levels during both basal and potentiated synaptic transmission.  Ref: 10456087 Learn MemLearn Mem, 2000
- These results suggest that  **PKC**  may couple the postsynaptic mGluR5 to the NMDA receptor potentiation during  **LTP** ,.  Ref: 10454358 Learn MemLearn Mem, 2000
- RATIONALE The hippocampal protein kinase C  **PKC**  family is involved in the early events of consolidation of long term potentiation  [ **LTP** ]   **LTP**  and long term memory LTM.  Ref: 10867979 Psychopharmacology (Berl), 2000
- **PKC**  potentiation of NMDAR function is likely to be an important mode of NMDAR regulation in vivo and may play a role in NMDA dependent long term potentiation  [ **LTP** ] .  Ref: 10611373 Proc Natl Acad Sci U S A, 1999
- Several evidences demonstrate that protein kinase C  **PKC**  is involved in hippocampal long term potentiation  [ **LTP** ]   **LTP**  and in different forms of learning, including inhibitory avoidance training in rats.  Ref: 10677591 Brain Res, 2000
- Moreover, the anoxic  **LTP**  was completely abolished in neurons intracellularly recorded with the selective  **PKC**  inhibitor PKCI 19 36 50 microM.  Ref: 9855311 Neuroreport, 1998
- We show that inhibiting postsynaptic calcium CaM dependent protein kinase II CaM KII and calcium phospholipitidyserine dependent protein kinase  **PKC**  in hippocampal CA1 neurons attenuates significantly the expression of  **LTP** , but not basal synaptic transmission.  Ref: 10456087 Learn MemLearn Mem, 1998

**Parallel relationship** (e.g. studied together, co-existance, homology, etc.)

- Activation of protein kinase C  **PKC**  is one of the biochemical pathways thought to be activated during activity dependent synaptic plasticity in the brain, and long term potentiation  [ **LTP** ]   **LTP**  and long term depression LTD are two of the most extensively studied models of synaptic plasticity.  Ref: 10537078 J Neurochem, 1999
- ... the action is independent of NMDA receptors, induces an  **LTP**  like facilitation of hippocampal synaptic transmission as a consequence of modulation of nicotinic ACh receptors and  **PKC** .  Ref: 10224305 Brain Res, 1999
- In particular, we have investigated the possible role of  **PKC**  in a molecular switch process that is triggered by the synaptic activation of metabotropic glutamate receptors and regulates the induction of  **LTP** .  Ref: 11069602 Eur J Neurosci, 2000
- In the present study we have examined the role of  **PKC**  in tetanus induced  **LTP**  of AMPA receptor mediated synaptic transmission in hippocampal slices obtained from adult rats.  Ref: 11069602 Eur J Neurosci, 2000
- These results suggest that the increase of calcium influx through NMDA channels and L type VOCCs in turn triggering a  **PKC**  dependent signaling cascade is a possible cellular basis underlying this seizure like activity induced inhibition of  **LTP** .  Ref: 10980002 J Neurophysiol, 2000
- Our results are consistent with the hypothesis that the effects of lead on  **LTP**  are mediated via an action on  **PKC** , which varies with age.  Ref: 10876039 Brain Res Dev Brain Res, 2000
- The results of the present study thus suggest that arachidonic acid sustains enhanced activity of nicotinic ACh receptors by interacting with a  **PKC**  pathway, thereby increasing glutamate release from presynaptic terminals, and then leading to an  **LTP**  like facilitation of hippocampal synaptic transmission.  Ref: 10366747 Brain Res Mol Brain Res, 1999
- Transient removal of extracellular magnesium elicits persistent suppression of  **LTP**  at hippocampal CA1 synapses via  **PKC**  activation.  Ref: 10980002 J Neurophysiol, 2000
- The presence of cPLA2 and  **PKC**  in hindbrain suggests that these enzymes play a central role in neurotransmitter release, long term potentiation  [ **LTP** ]  and neuritogenesis in this area under normal conditions.  Ref: 10536872 J Hirnforsch, 1999
- Here we have examined changes in the in situ phosphorylation level of two major  **PKC**  substrates, myristoylated alanine rich C kinase substrate MARCKS and growth associated protein GAP 43 B 50, after pharmacological stimulation or induction of  **LTP**  or LTD in the CA1 field of the hippocampus.  Ref: 10537078 J Neurochem, 1999
